# Making diagnostic tests as essential as medicines

**DOI:** 10.1136/bmjgh-2018-001033

**Published:** 2018-08-03

**Authors:** Adriana Velazquez Berumen, Sarah Garner, Suzanne Rose Hill, Soumya Swaminathan

**Affiliations:** 1 Department of Essential Medicines and Health Products, World Health Organization, Geneva, Switzerland; 2 Office of Deputy Director General for Programmes, World Health Organization, Geneva, Switzerland

**Keywords:** diagnostics and tools, health systems, public health, viral hepatitis

The World Health Organization (WHO) has launched the first edition of the Model List of Essential in vitro Diagnostics (EDL),^1^ an initiative that we hope will prove to be as important as the launch of Essential Medicines List 45 years ago. The global health community has focused on access to health products for prevention (vaccines) and treatment (medicines) over the last 20 years, but despite the consequences of underdiagnosis and misdiagnosis (including mistreatment, health complications and costly unnecessary interventions), there has been very little focus in the global health dialogue to date on access to appropriate diagnostic tests to ensure appropriate treatment. In high-income countries, tests are generally available through clinical laboratories but in low-income and middle-income settings, these services are not accessible, so more focus needs to be on reliable tests for community settings.

It is clear that without quality-assured diagnostic tests, the practice of medicine is blind and curable conditions may not be treated. As one example, hepatitis C virus (HCV) affects an estimated 71 million people worldwide, but it is estimated that only 20% have been diagnosed.^2^ It can be cured with a 3-month course of treatment that now is affordable in many low-income and middle-income countries and that can be given in community settings. Yet in many countries, testing for hepatitis Cis restricted to specialised testing laboratories although there are now rapid oral fluid tests^3^ that can be used to self-test,^4^ thus avoiding concerns of stigma as well as potentially being widely available. Several other examples were also raised in Schroeder *et al*^5^ and a long list is clearly possible.

The world has agreed that health coverage should be universal. The Sustainable Development Goal (SDG) 3.8 sets the following target for 2030: Achieve universal health coverage (UHC), including financial risk protection, access to quality essential healthcare services and access to safe, effective, quality and affordable essential medicines and vaccines for all.^6^ Progress towards UHC means a lowering of barriers to seeking and receiving needed care: for example, out-of-pocket payments, distance, poorly equipped facilities and poorly trained health workers and receive the interventions currently agreed to be necessary.^7^ Access to diagnostic tests has to be a key part of this goal.

The first edition of the EDL starts with two categories of in vitro diagnostics (IVDs)—general and disease specific. These recommendations are drawn from existing WHO guidelines, manuals and priority lists of medical devices that have been supported by a review of the evidence.^8 9^ The EDL includes basic information on the test: if it is for general routine assessment or specific to a disease, the purpose of the test, the assay format, type of sample, links to relevant WHO documents, or to any WHO prequalified or endorsed products.

The general IVDs include those for clinical chemistry, blood transfusion, serology, microbiology, mycology, parasitology and haematology. These tests form the basic IVDs package to support routine diagnosis and monitoring of many conditions, such as diabetes, cardiovascular, anaemia and liver function. The disease-specific IVDs reflect the existing global priority disease priorities on WHO work programme; HIV,^10^ Hepatitis B and C,^11^ human papilloma virus,^12^ malaria,^13^ syphilis^14^ and tuberculosis.^15^

For each IVD, it was recommended that guidance should be given to member states as to which level of care the test is most suited to; primary care or more specialised facilities, according to level of the facilities and expertise required. Primary healthcare tests can be used in mobile units, emergency situations, at home, community level and includes self-testing products. Other tests are more suited to clinical laboratories that require appropriately trained healthcare workers. The EDL itself will be expanded in subsequent editions to include IVDs required for non-communicable diseases, neglected tropical diseases and those required for emergencies and outbreaks. The inclusion of IVDs to support global efforts to combat antimicrobial resistance is a specific priority.

The creation of the EDL is the first step in ensuring that all communities everywhere can benefit from diagnostic technology as a key component for achieving UHC. But implementation in countries will be critical. While some Member States already have national lists of medical technologies/devices (57% of 173 countries), others still not.^16^As with the Model List of Essential Medicines, we expect that countries will have to adapt the EDL to suit their needs and systems, according to their local settings, work force, facilities, epidemiology and budgets. We also hope that it can support more efficient procurement and affordable prices.

Why has it taken so long to develop an EDL, given that the Essential Medicines List has been around for 40 years? Part of the answer to this question lies in the recognition of the technological advances in relation to point of care diagnostics and rapid tests, that now mean that it is in fact possible to self test for HIV, for example. It is also due to the increasing number of medicines that are being developed for targeted populations, such as trastuzumab for HER 2 positive breast cancer, where the treatment is in ineffective or harmful if given to the wrong subgroups of patients. The global actions on anti-microbial resistance (AMR), and the need to be able to diagnose the cause of infections accurately to improve use of antibiotics is another factor, as are the emergency responses required over the past several years—to Ebola, Zika and now Nipah virus outbreaks—that have already created pressure for accurate diagnostics.

But whatever the reasons for not doing it sooner, the challenge now is to use the interest and momentum that this first list creates, to make a difference to patients. We know for medicines that despite 40 years of a model list, a third of the patients around the world still do not have access to the medicines needed—hopefully, we can make a difference much more quickly for diagnostics.
